# *e*Thread: A Highly Optimized Machine Learning-Based Approach to Meta-Threading and the Modeling of Protein Tertiary Structures

**DOI:** 10.1371/journal.pone.0050200

**Published:** 2012-11-21

**Authors:** Michal Brylinski, Daswanth Lingam

**Affiliations:** 1 Department of Biological Sciences, Louisiana State University, Baton Rouge, Louisiana, United States of America; 2 Center for Computation & Technology, Louisiana State University, Baton Rouge, Louisiana, United States of America; 3 Department of Electrical and Computer Engineering, Louisiana State University, Baton Rouge, Louisiana, United States of America; University of Alberta, Canada

## Abstract

Template-based modeling that employs various meta-threading techniques is currently the most accurate, and consequently the most commonly used, approach for protein structure prediction. Despite the evident progress in this field, accurate structure models cannot be constructed for a significant fraction of gene products, thus the development of new algorithms is required. Here, we describe the development, optimization and large-scale benchmarking of *e*Thread, a highly accurate meta-threading procedure for the identification of structural templates and the construction of corresponding target-to-template alignments. *e*Thread integrates ten state-of-the-art threading/fold recognition algorithms in a local environment and extensively uses various machine learning techniques to carry out fully automated template-based protein structure modeling. Tertiary structure prediction employs two protocols based on widely used modeling algorithms: Modeller and TASSER-Lite. As a part of *e*Thread, we also developed *e*Contact, which is a Bayesian classifier for the prediction of inter-residue contacts and *e*Rank, which effectively ranks generated multiple protein models and provides reliable confidence estimates as structure quality assessment. Excluding closely related templates from the modeling process, *e*Thread generates models, which are correct at the fold level, for >80% of the targets; 40–50% of the constructed models are of a very high quality, which would be considered accurate at the family level. Furthermore, in large-scale benchmarking, we compare the performance of *e*Thread to several alternative methods commonly used in protein structure prediction. Finally, we estimate the upper bound for this type of approach and discuss the directions towards further improvements.

## Introduction

With the continuing advances in genome sequencing [1], there has been a rapid accumulation of protein sequences, whose structures are yet to be annotated. As of October 2012, there are >1.7×10^7^ unique protein sequences from 17,994 organisms in the Reference Sequence database [2]. However, due to low-sequence identity to already annotated proteins, the molecular functions of many of these gene products remain unknown. Using standard homology-based tools poses a significant risk associated with the “overprediction” of molecular function and, as an inevitable consequence, typically results in high levels of misannotation [3]. On that account, more accurate and confident function annotation tools are needed; here structure-based approaches show a considerable promise [4]. Early methods for function inference from protein structure were very sensitive to the quality of the target structures and typically required these solved experimentally by X-ray crystallography or NMR. More recent approaches are generally devoid of these limitations and can routinely annotate low-to-moderate quality protein models [5], [6], [7], [8]. Consequently, protein structure modeling plays an important role in Functional Genomics by providing structural information on gene products that is subsequently utilized by powerful structure-based approaches to protein function inference [9], [10].

Currently, the most accurate and the most widely used methods for protein structure prediction build on homology, i.e. they use information educed from related proteins. As demonstrated in the recent community-wide Critical Assessment of Protein Structure Prediction (CASP) experiment, the top performing groups in tertiary structure prediction category used various template-based methods [11]. One of the best algorithms in the field, I-TASSER, builds three-dimensional models from multiple-threading alignments constructed by LOMETS [12] using iterative assembly/refinement simulations [5]; this is followed by function prediction by matching the models to proteins with known functions [5]. Another development from this prolific group is QUARK, a method for protein structure assembly using continuous template fragments [13]. QUARK first identifies small structural fragments by gapless threading against the Protein Data Bank (PDB) [14] and then ranks them using a composite scoring function, which consists of sequence and structure profiles, predicted secondary structure and backbone torsion angles. For each position in the target, the top-scored fragments are used to assemble a 3D model by Replica Exchange Monte Carlo simulations. Recent improvements of template selection methods include the development of HHblits, a new iterative HMM-HMM sequence search algorithm [15]. HHblits was demonstrated to have 50–100% higher sensitivity than PSI-BLAST [16] and to produce multiple alignments of much higher quality. Furthermore, advances in the quality assessment protocol result in a significant gain in the overall performance of IntFOLD-TS, which first generates a large number of alternate models using in-house versions of several different alignment methods and then ranks them in terms of the estimated global quality [17]. Importantly, highly accurate predictions of local errors, provided in the resulting models, make this method useful for guiding future experimental work. Improved prediction of secondary structure, backbone torsion angles and solvent accessible surface area significantly increases the accuracy of SPARKS-X, which is one of the best single-method fold recognition techniques [18]. Finally, RaptorX uses a novel statistical learning model and a multiple-template threading component to provide better measure of the compatibility between the target sequence and the template structures [19]. Indeed, the constructed alignments are much more accurate than those built by its predecessor, RAPTOR. These and many other successful examples show that there is an encouraging progress in this field, which certainly will have impact on many areas of modern molecular and cell biology.

Notwithstanding the success of single-threading approaches, meta-threading techniques are the ones that make headway in protein structure prediction. These methods identify template structures and construct target-to-template alignments by considering outputs from a variety of individual threading algorithms. Typically, the combined predictions have a higher chance to be accurate than those produced by a single method. Recent CASP experiments demonstrated that models generated from predictions by meta-threading servers are more accurate than the best individual server alone [11], [20]. Moreover, an important additional advantage of meta-predictors is the improved estimation of the reliability of predictions. An example of such a successful meta-server is LOMETS, which currently uses ten threading algorithms to generate initial structural models and constraints for the prediction of protein tertiary structures [12]. Models in LOMETS are selected from individual programs purely based on consensus, i.e. the structure similarity of the considered model with other threading alignments. The consensus predictions provided by LOMETS were shown to be more accurate than those generated by individual component methods. Another example is Pcons, a neural-network–based consensus predictor that improves fold recognition by selecting the best model out of those produced by six prediction servers [21]. Pcons translates the confidence scores reported by each server into uniformly scaled values corresponding to the expected accuracy of each model. The translated scores as well as the similarity between models produced by different servers are used in the final selection. According to benchmarks carried out for two unrelated sets of newly solved proteins, Pcons outperforms any single server.

In this communication, we describe *e*Thread, a highly accurate meta-threading procedure to identify templates for the template-based modeling of protein structures. This new method uses ten state-of-the-art threading algorithms and machine learning designed specifically for the optimal selection of structure templates. In large-scale benchmarks, we demonstrate that the performance of *e*Thread in the identification of structurally related templates is notably higher than any of the individual single-threading algorithms. Template-based protein structure modeling requires not only a set of structure templates but also the corresponding target-to-template alignments and/or predicted inter-residue contacts. Therefore, as a part of *e*Thread software, we developed a new machine learning procedure to combine alignments reported by individual meta-threading algorithms into a set of consensus alignments. We also developed *e*Contact, a Bayesian classifier with an optimized Gaussian kernel for the prediction of inter-residue contacts. Optimized sets of templates and the corresponding alignments as well as predicted long-range contacts are integrated into structure assembly protocols for the construction of full-length models of the target proteins. Two separate procedures have been devised based on widely used modeling algorithms: Modeller [22] and TASSER-Lite [23]. In addition, we designed *e*Rank, which effectively ranks generated multiple protein models and provides reliable confidence estimates for structure quality assessment. To demonstrate the utility of this approach, modeling protocols were optimized and carefully benchmarked on a large and representative dataset of protein structures and compared to the performance of several alternative methods commonly used in protein structure prediction. Finally, we estimate the upper bound for this type of approach and discuss the directions towards further improvements. *e*Thread webserver as well as benchmarking datasets and results are freely available to the academic community at http://www.brylinski.org/ethread.

## Materials and Methods

### Method Overview

**Figure 1:**
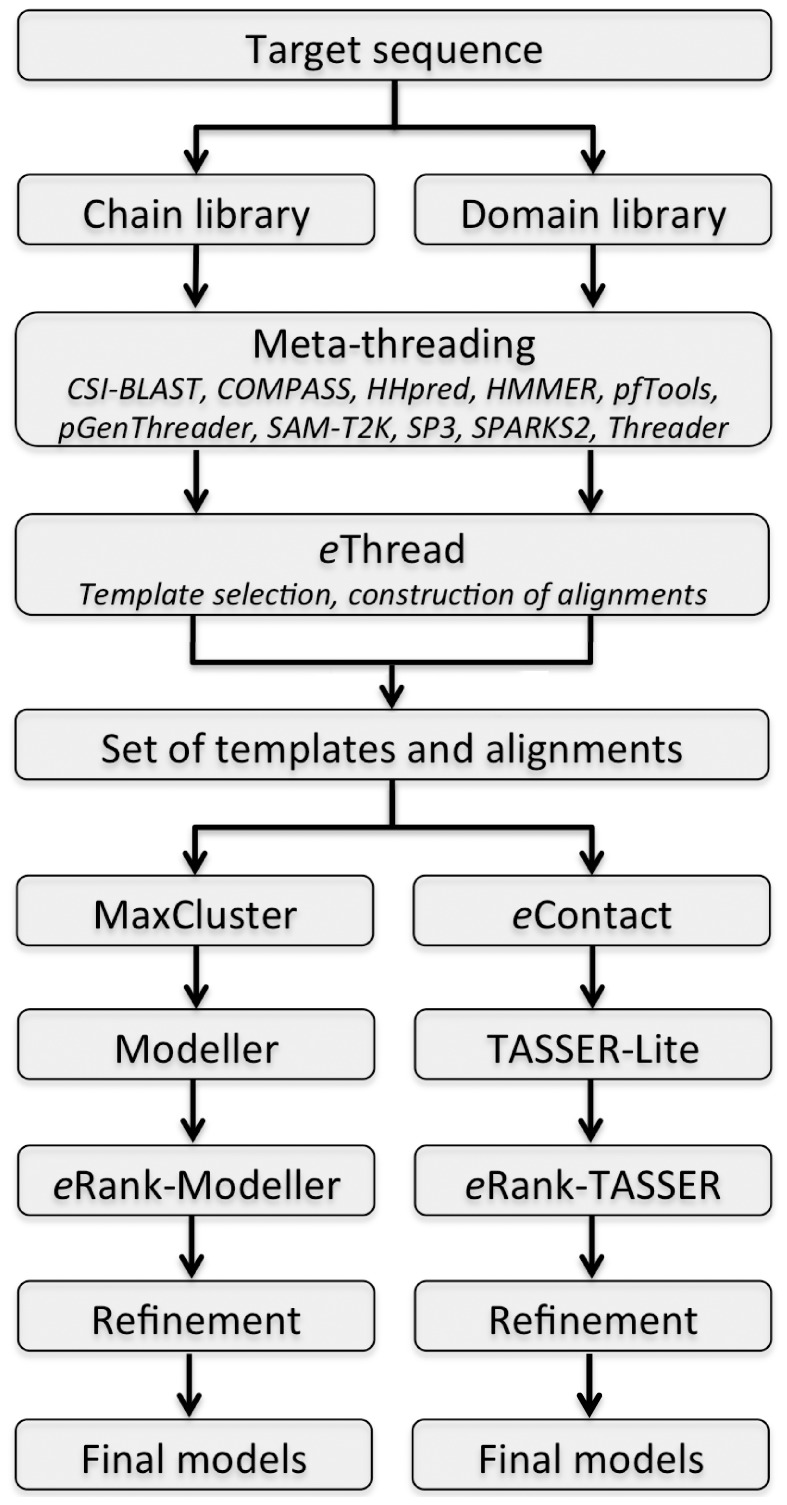
Flowchart of meta-threading using *e*Thread. Modeling stages include template selection, alignment construction, inter-residue contact prediction, 3D structure modeling, and model ranking. The details are given in text.

A flowchart for the *e*Thread algorithm is shown in Figure 1. For a given amino acid sequence, the method starts by applying meta-threading to search for structurally similar templates in two libraries, which consist of full protein chains as well as individual domains. The inclusion of individual domains is a commonly used practice in threading to improve the recognition of those templates that may only partially cover a multiple-domain target [24]. In addition, if a full chain template is found, it also provides the information on the mutual orientation of domains. The identified templates are subsequently filtered by *e*Thread and the corresponding target-to-template alignments are constructed. Next, two structure modeling protocols are used to build the three-dimensional models of the target: Modeller [22], which employs template pre-clustering by MaxCluster, and TASSER-Lite [23], which additionally incorporates inter-residue contacts predicted by *e*Contact. In both cases, the resulting models are ranked by *e*Rank, assigned confidence estimates, and refined using molecular mechanics. Below is a detailed description of the benchmarking dataset as well as the individual modeling stages.

### Dataset

Benchmarking dataset was compiled from all PDB entries as of Jan 2012. Using PISCES [25] to remove redundancy at the 40% sequence identity resulted in 11,468 representative protein chains 50–600 residues in length. Furthermore, we excluded 2,596 proteins, for which no structurally related proteins can be detected using any of the individual threading component methods. The final dataset consists of 8,872 non-redundant and representative protein targets.

### Threading Libraries

Two threading libraries are used in this study: chain and domain. Chain library comprises aforementioned 11,468 protein chains selected from the PDB by PISCES [25]. Domain library was compiled by PISCES using the Structural Classification of Proteins (SCOP) database [26]. Similarly to the chain library, the redundancy was removed at 40% pairwise sequence identity. This library contains 10,013 representative protein domains 50–600 residues in length, for which the atomic coordinates were obtained from the ASTRAL database [27].

### Threading Component Methods and Template Selection

*e*Thread is a meta-threading procedure, which integrates ten state-of-the-art protein threading/fold recognition algorithms: CSI-BLAST [28], COMPASS [29], HHpred [30], HMMER [31], pfTools [32], pGenThreader [33], SAM-T2K [34], SP3 [24], SPARKS2 [24] and Threader [35]. Each individual threading/fold recognition algorithm assesses structures present in the template library using some scoring system, e.g. SP3, SPARKS2 and Threader assign Z-scores using the entire template library as a background, COMPASS, CSI-BLAST, HMMER and SAM-T2K employ scoring systems based on analytically estimated E-values, and HHpred uses calibrated probabilities for true relationships between proteins. For the template selection, we constructed a machine learning model based on feature vectors composed of individual threading scores. The machine learning employs Support Vector Machines for classification problems (SVC) [36] to assess whether a particular template is structurally related to the target with a TM-score [37] of ≥0.4. The accuracy of template selection is assessed using 2-fold cross validation excluding those templates, whose sequence identity to target is >40%. We note that this sequence identity cutoff is also applied in all subsequent modeling steps.

### Consensus Target-to-template Alignments

As a part of *e*Thread, we also developed a new machine learning procedure to combine alignments reported by individual meta-threading algorithms into a set of consensus alignments. Specifically, we built a Naïve Bayes classifier, which was trained on meta-threading data against reference structure alignments constructed by fr-TM-align [38]. First, from individual alignments produced by the component methods, this model estimates the posterior probability of each pair of residues to be a part of the target-to-template structure alignment. Subsequently, the matrix of Bayesian probabilities is used as a scoring function in Needleman-Wunsch Dynamic Programming (DP) [39] to construct the consensus global alignments. Similarly to the template selection, the consensus alignment model is assessed using 2-fold cross validation.

### Inter-residue Contact Prediction

Long-range contacts between residues are defined when a pair of their heavy atoms is within a distance of 4.5 Å and they are separated in the sequence by at least 4 other residues. Inter-residue contacts are predicted from consensus target-to-template alignments by *e*Contact, a machine learning approach. For a pair of residues, we calculate a vector of four features: the fraction of templates that have residues in equivalent positions in contact with each other, the average confidence of these templates that have such contacting residues, and the average confidence of the corresponding target-to-template alignments; in addition, we also include a knowledge-based statistical pair potential [40]. Based on these feature vectors, a SVC [36] model was constructed to estimate the probability of a given pair of residues to be in contact. The accuracy was assessed by 2-fold cross validation.

### Tertiary Structure Modeling

To construct three-dimensional models of the target proteins, we employ two commonly used template-based modeling algorithms: Modeller [22] and TASSER-Lite [23]. Both programs use threading alignments generated by *e*Thread as input. In addition, TASSER-Lite also uses inter-residue contacts predicted by *e*Contact. For Modeller, the set of templates identified by *e*Thread is pre-clustered by MaxCluster (http://www.sbg.bio.ic.ac.uk/maxcluster/) using a TM-score clustering threshold of 0.4 and the models are subsequently constructed individually for each cluster. The side chains in the structures modeled by Modeller and TASSER-Lite are rebuilt from the Cα trace by Pulchra [41] and finally, all-atom structures are refined in the CHARMM22 force field [42] using the Jackal modeling package [43].

### Model Ranking and Confidence Estimates

Both Modeller and TASSER typically generate multiple models for a given target. To rank the resulting models and to assign confidence estimates, we developed *e*Rank that employs SVM-Rank, a version of Support Vector Machines designed specifically for ranking problems [44]. *e*Rank also estimates the TM-score to native using Support Vector Regression (SVR) [36]. Both ranking and confidence estimate models use the following set of features: the confidence of alignments constructed by *e*Thread (*Alignment*), the average alignment coverage (*Coverage*), DOPE score [22] (*DOPE*), dFire residue-level potential of mean force [45] (*dFire*), secondary structure match between the model and the PSIPRED [46] prediction (*PSIPRED*), burial score (*Burial*, see Text S1) and secondary structure preferences (*SecStr*, see Text S1). In addition, *e*Rank/Modeller includes the fraction of templates assigned to a particular cluster by MaxCluster (*Fraction*) and the GA341 score [22] (*GA341*). *e*Rank/TASSER-Lite also incorporates the average TM-score of a given model to templates identified by *e*Thread (*TM-score*, roughly equivalent to *Fraction* for Modeller) as well as the following clustering coefficients reported by SPICKER [47], which is a part of the TASSER-Lite suite: cluster fraction (*TASSER-Lite^fract^*), cluster density (*TASSER-Lite^dens^*) and cluster mean energy (*TASSER-Lite^ene^*). Both ranking ability and the accuracy of confidence estimates are assessed using 2-fold cross validation.

### Other Approaches to Structure Modeling

We compare the accuracy of *e*Thread models to those constructed by two alternative protocols. The first one is a naïve, single-template approach: For a given target sequence, we run 5 iterations of PSI-BLAST [16] to identify weakly homologous proteins and we select the top-ranked as the structure template. A three-dimensional model is then constructed by Nest [43] using the target-to-template alignment provided by PSI-BLAST. The resulting model is additionally subject to all-atom structure refinement using the Jackal modeling package [43]. The second approach represents a single-threading, multiple-template algorithm; here, we use the original implementation of TASSER-Lite [23]. For both PSI-BLAST/Nest and TASSER-Lite, we exclude from the modeling procedure all closely related templates with >40% sequence identity to the target in order to make the results comparable to those obtained by *e*Thread-based modeling.

## Results

### Template Identification

**Figure 2:**
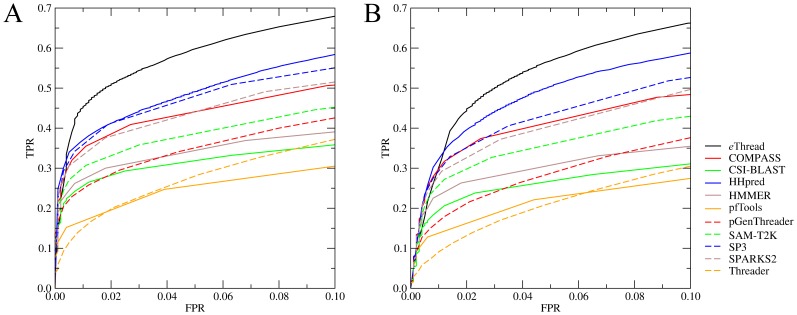
ROC plots for the identification of structurally similar templates. Template structures are selected from (**A**) chain and (**B**) domain library.

The ability of a threading algorithm to select those templates that are structurally similar to the target is critical for the subsequent construction of three-dimensional models. Here, we define a good template as the structure with a statistically significant TM-score [37] to native of ≥0.4. We note that a TM-score of 0.4 is an appropriate fold similarity assignment threshold; template structures above this value contain sufficient information to enable the full-length reconstruction of the target structure [48]. TM-score is calculated by fr-TM-align [38] for both threading libraries used in this study. Trivial templates with more than 40% sequence identity to target are excluded from this as well as all subsequent analyses. Figure 2 shows ROC plots for *e*Thread compared to the individual threading component methods. The accuracy does not depend on the library used (Figure 2A – chain, Figure 2B – domain); however, it varies across different algorithms. HHpred was found the most accurate single method with a true positive rate (TPR) of 0.49/0.50 at the expense of 0.05 false positive rate (FPR) for the chain/domain library. At the same FPR, the next accurate algorithms: SP3, COMPASS and SPARKS2 give a TPR of 0.47/0.43, 0.44/0.41 and 0.44/0.40, respectively. However, the effective combination of multiple algorithms considerably extends the coverage of target sequences by distantly related templates and increases the true positive rate; the corresponding TPR values for *e*Thread are 0.60/0.57 (at 0.05 FPR). Thus *e*Thread systematically detects more structure templates than any of the component methods. The probability values returned by machine learning also contribute to the overall modeling confidence.

### Quality of Threading Alignments

**Figure 3:**
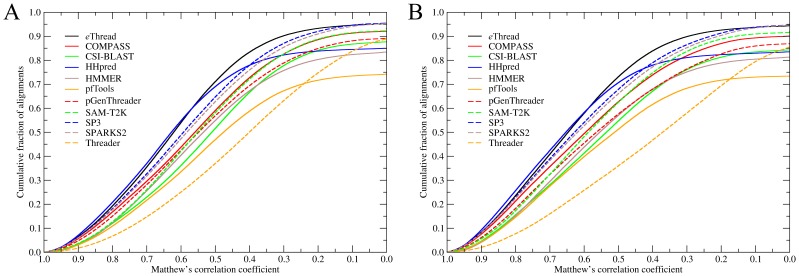
Accuracy of threading target-to-template alignments. The accuracy is assessed by Matthew’s correlation coefficient against structure alignments for (**A**) chain and (**B**) domain library.

Effective template selection is still not sufficient for practical applications, such as protein structure modeling. In addition, target-to-template alignments should also be accurate to build a correct model. In Figure 3, we assess the quality of threading alignments constructed by *e*Thread as well as all component methods by Matthew’s correlation coefficient (MCC) against structure alignments by fr-TM-align. Again, HHpred, SP3 and SPARKS2 were found to be the most effective single-threading algorithms that build alignments with a MCC of ≥0.5 for the chain (domain) library for 69% (71%), 65% (68%) and 63% (66%) of the targets, respectively. The performance of *e*Thread is slightly lower than that of HHpred for MCC>0.6; however, it still provides good quality alignments in the MCC range of 0.4–0.6 for additional 4–8% of the targets, on average.

### Accuracy of Inter-residue Contacts

**Figure 4:**
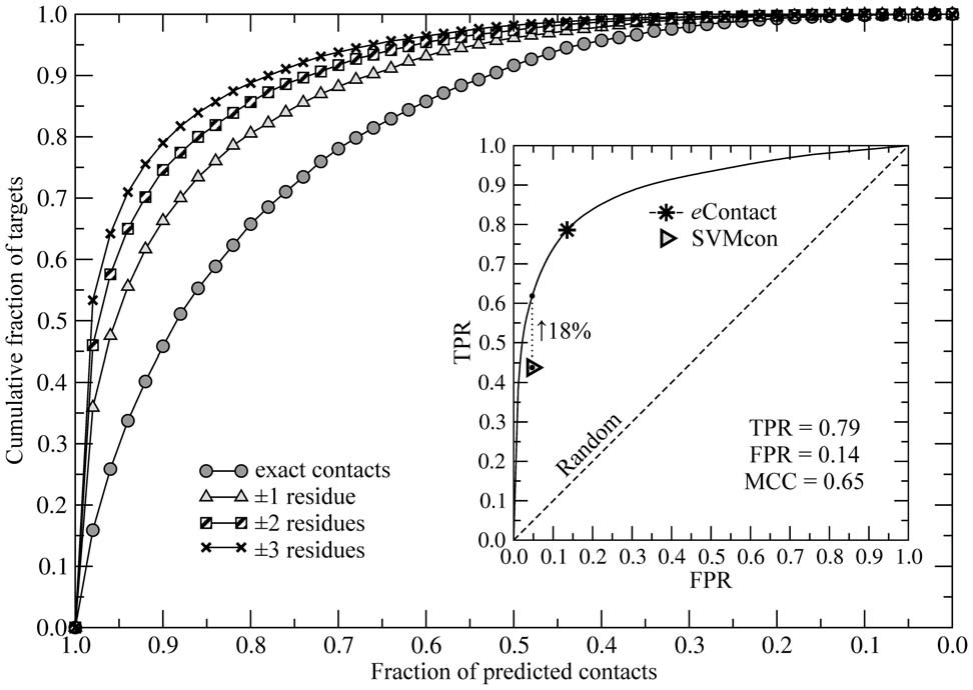
Accuracy of inter-residue contact prediction. The accuracy is evaluated for exact contacts as well as those within 1, 2 and 3 residues from the exact contact. Inset: ROC plot for the contact prediction; TPR – true positive rate, FPR – false positive rate. Star corresponds to a contact probability that maximizes MCC for *e*Contact, gray triangle depicts the performance of SVMcon and the dotted line shows the TPR improvement of *e*Contact over SVMcon for the FPR fixed at 0.047.

In addition to threading templates and target-to-template alignments, TASSER-Lite also incorporates predicted inter-residue contacts as an important component of its force field [49]. Here, we developed *e*Contact, a machine learning-based method for the prediction of long-range contacts. *e*Contact uses threading alignments as well as a generic knowledge-based pair potential; its cross-validated performance on a representative dataset is shown in Figure 4. At least 75% of exact native contacts are recovered for 72% of the target proteins. To select the optimal cutoff value for contact prediction, we use MCC, which represents a balanced measure that can be used if the classes are of different sizes [50]. The contact probability threshold of 0.35 maximizes MCC to 0.65 against the exact native contacts and yields 0.79 of true positive rate at the expense of only 0.14 false positives (Figure 4 inset). The accuracy further increases, when contacts within 1, 2 and 3 residues are also considered positives. Here, the fraction of targets with ≥75% of predicted native contacts is 84%, 88% and 91%, respectively. We note that TASSER-Lite, which employs low-resolution modeling, can effectively accommodate inter-residue contacts slightly mispredicted by a couple of residues.

We also compare the performance of *e*Contact to that of SVMcon, ranked as one of the top residue contact predictors in CASP7 [51]. SVMcon employs machine learning and a set of features, which include sequence profiles, secondary structure, solvent accessibility and contact potentials [52]. At a fixed FPR rate of 0.047, *e*Contact and SVMcon yield TPR of 0.62 and 0.44, respectively (Figure 4 inset); thus *e*Contact predicts 18% more contacts than SVMcon.

### Ranking Ability

**Figure 5:**
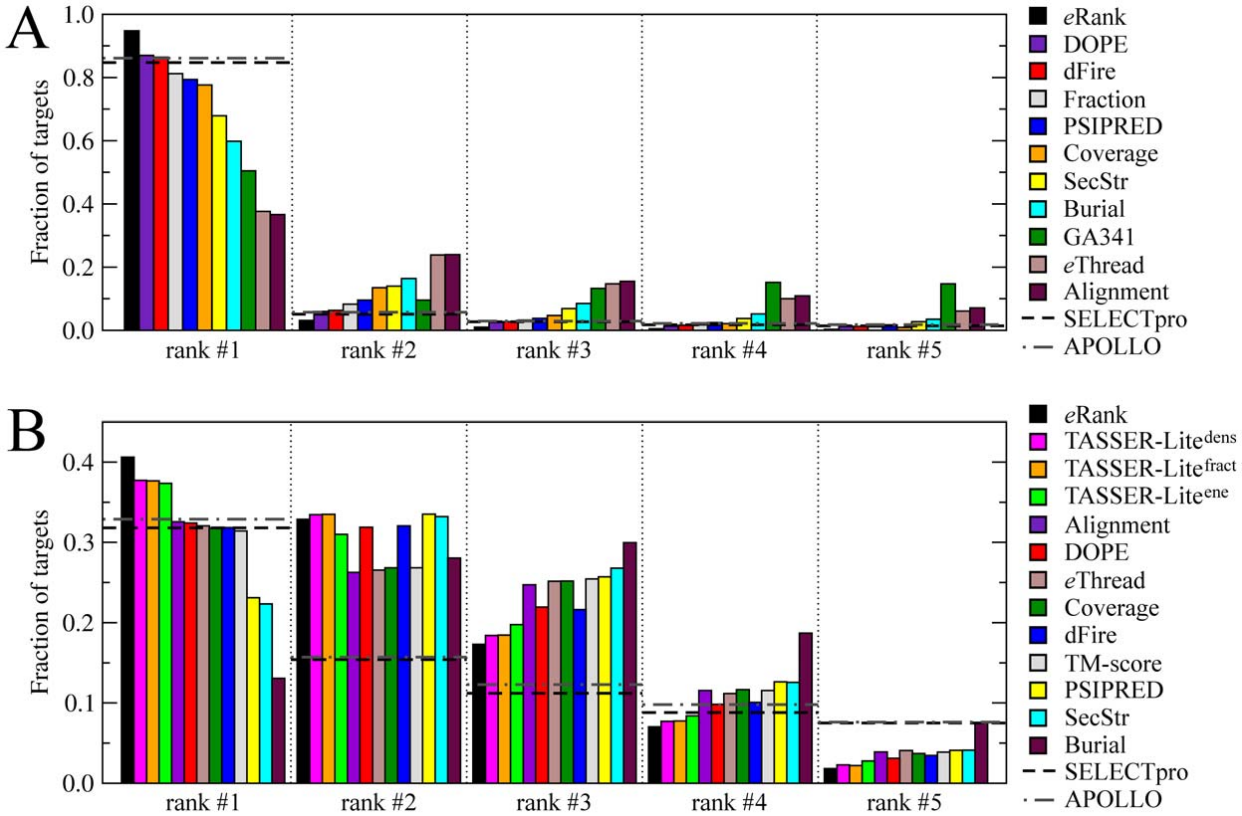
Ranking accuracy by *e*Rank. Structure models constructed by (**A**) Modeller and (**B**) TASSER-Lite are ranked and the corresponding accuracy is assessed by the fraction of targets for which the best models was found at a particular rank. Dashed black and dotted/dashed gray line depicts ranking accuracy by SELECTpro and APOLLO, respectively.

Both Modeller and TASSER-Lite typically build multiple models. For Modeller, we first pre-cluster the set of templates identified by *e*Thread and then construct a structural model for each cluster. TASSER-Lite generates Monte Carlo trajectories, which are subsequently clustered by SPICKER and a structure closest to the cluster centroid is selected for each cluster. To select the best models, we developed *e*Rank/Modeller and *e*Rank/TASSER-Lite; both are machine learning approaches that use a variety of scoring functions. In Figure 5, we assess the ranking ability of *e*Rank, i.e. in how many cases the best model is found amongst the top 5 ranks; we also compare the performance of *e*Rank to the component scoring functions. We note that the “best model” may not be necessarily highly accurate; it is just better than the other models constructed. As shown in Figure 5A, *e*Rank/Modeller correctly identifies the best model in 95% of the cases, which represents an improvement over the most effective individual scoring terms: *DOPE* (87%), *dFire* (86%), *Fraction* (81%), *PSIPRED* (79%) and *Coverage* (78%). *e*Rank/TASSER-Lite ranks the best model as the 1^st^, 2^nd^ and 3^rd^ one in 41%, 33% and 17% of the cases, respectively (see Figure 5B). Again, this ranking accuracy is higher than *TASSER-Lite^dens^*, *TASSER-Lite^fract^* and *TASSER-Lite^ene^*, which place the best model at rank 1 for 38%, 38% and 37% of the targets, respectively.

In Figure 5, *e*Rank is also compared to SELECTpro [53] (dashed black) and APOLLO [54] (dotted/dashed gray), which are structure-based model selection methods. SELECTpro uses a sophisticated energy function that comprises physical, statistical and predicted structural scoring terms and was shown in large-scale benchmarks to be highly effective. APOLLO evaluates the absolute single and pair-wise global structure quality in terms of the GDT-score [55]; here, we use the single-model approach. *e*Rank outperforms SELECTpro for the models constructed by Modeller (TASSER-Lite) by ∼10%; here, the best model is assigned rank 1 in 95% (41%) and 85% (32%) of the cases, respectively. The accuracy of APOLLO is slightly higher than SELECTpro; however, ∼8% worse than *e*Rank: the best *e*Thread/Modeller (*e*Thread/TASSER-Lite) model is assigned rank 1 in 86% (33%) of the cases. We note that *e*Rank was specifically tailored to structure modeling using *e*Thread, whereas SELECTpro and APOLLO represent general quality assessment approaches, applicable to any set of protein models. The difference in performance between *e*Rank/Modeller and *e*Rank/TASSER-Lite comes from the way models are constructed and from the pairwise similarities between the top-ranked structures. Multiple TASSER-Lite models are often structurally similar to each other (as well as to the target), thus the ranking is more difficult. The pre-clustering procedure used in the model construction by Modeller typically results in a set of very different structures with a pairwise TM-score of <0.4; consequently, at most one model would be structurally similar to the target.

### Model Accuracy

We use TM-score [37] to native as the main assessment metric for the accuracy of the top-ranked models. Note that the TM-score is a protein length independent measure of structural similarity with a statistical significance at ≥0.4. In addition, we assess the structure quality using several other well established measures: Cα-RMSD [56], Gaussian-weighted RMSD (wRMSD) [57], MaxSub [58] and GDT-score [55]. Benchmarking results reported here were obtained for a non-redundant and representative subset of the PDB; therefore are easily comparable to other studies that use a similar setup. Moreover, these statistics provide reliable estimates of the expected accuracy in large-scale applications, e.g. genome-wide protein structure modeling projects [59].

**Figure 6:**
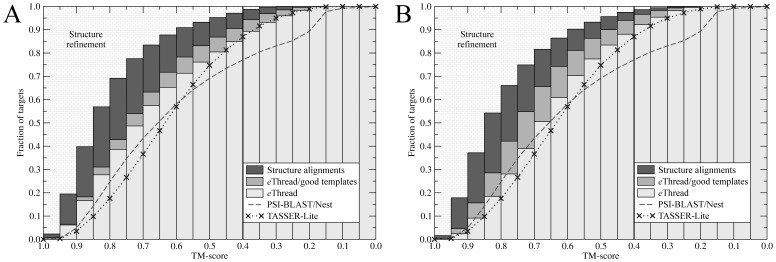
Global quality of protein models assessed by the TM-score to native. Three sets of (**A**) Modeller and (**B**) TASSER-Lite models are constructed using: structure alignments, *e*Thread alignments for structurally related (“good”) templates only as well as all alignments generated by *e*Thread. Gray-bricked area points up a room for further improvement using structure refinement. Dashed and dotted line corresponds to the accuracy of PSI-BLAST/Nest and the original TASSER-Lite, respectively.

For both Modeller and TASSER-Lite, we also evaluate the models constructed using 3 different protocols to ascertain, where the future improvements are most likely to increase the overall accuracy of structure modeling. First, we assess the complete *e*Thread procedure, i.e. template identification, alignment construction and model assembly/refinement. Next, to evaluate the quality of target-to-template alignments, we include only these templates that are structurally related to the target with a TM-score of ≥0.4. Finally, we estimate the upper bound for the modeling accuracy using structurally similar templates only and the corresponding structure alignments constructed by fr-TM-align. The results for Modeller and TASSER-Lite are shown in Figure 6 as a fraction of targets whose structures are modeled to a given accuracy. Focusing on a high modeling accuracy at a TM-score of ≥0.7, the upper bound for the modeling protocols using Modeller and TASSER-Lite is 78% and 75%, respectively. The accuracy of modeling using *e*Thread alignments instead of these constructed by fr-TM-align (*e*Thread/good templates in Figure 6) decreases to 54% (by 24%) and to 55% (by 20%) for Modeller and TASSER-Lite, respectively. It shows that TASSER-Lite better accommodates alignment errors than Modeller. When the complete *e*Thread procedure is used, protein models with a TM-score of ≥0.7 are constructed by Modeller and TASSER-Lite for 49% and 39% of the targets, respectively. It demonstrates, that Modeller builds more highly accurate models; this is also shown in Table 1, which assesses the structure quality using several other measures. For example, the average MaxSub (GDT-score) for *e*Thread/Modeller and *e*Thread/TASSER-Lite is 0.55 (0.59) and 0.46 (0.50), respectively. However, Modeller provides slightly lower coverage of a dataset by models whose TM-score to native is ≥0.4 (still statistically significant) than TASSER-Lite: 85% and 88%, respectively (see Figure 6). Nevertheless, using *e*Thread identified templates and alignments and model ranking by *e*Rank, both structure modeling algorithms build correct (and often very high quality) models for a significant fraction of the benchmark proteins.

**Table 1:** Global structure quality of protein models.

Models	Cα-RMSD [Å]	wRMSD	TM-score	MaxSub-score	GDT-score
*e*Thread/Modeller	8.33±5.91	0.91±0.23	0.69±0.16	0.55±0.18	0.59±0.17
fr-TM-align/Modeller	6.03±5.24	0.83±0.22	0.78±0.15	0.66±0.17	0.68±0.16
*e*Thread/TASSER-Lite	9.40±6.51	1.00±0.25	0.63±0.18	0.46±0.19	0.50±0.19
fr-TM-align/TASSER-Lite	5.94±5.59	0.87±0.24	0.77±0.15	0.64±0.17	0.64±0.16
PSI-BLAST/Nest	10.99±6.66	0.90±0.22	0.60±0.24	0.48±0.23	0.51±0.23
TASSER-Lite	11.08±7.68	1.01±0.27	0.62±0.18	0.47±0.18	0.49±0.19

Models are constructed by Modeller and TASSER-Lite using *e*Thread alignments and compared to those built using structure alignments by fr-TM-align. The quality is assessed by several popular measures and additionally compared to that of a simple single-template approach, PSI-BLAST/Nest and a standard version of TASSER-Lite.

Mean values and the corresponding standard deviations are reported.

This modeling accuracy is also higher than that obtained using a simple single-template approach, see Figure 6 and Table 1. For 77% of the target proteins, PSI-BLAST/Nest constructs models whose TM-score to native is ≥0.4. This is 8% and 11% less than using *e*Thread/Modeller and *e*Thread/TASSER-Lite, respectively. When compared to a single-threading, multiple-template approach, the most notable improvement is for protein models with a TM-score to native of ≥0.7. Here, the original TASSER-Lite generates models with such accuracy for 37% of the targets, which is 2% less than using *e*Thread/TASSER-Lite; however, for 12% more target proteins high quality models are constructed using *e*Thread/Modeller. This justifies the computationally more expensive multiple-template modeling using meta-threading and *e*Thread.

### Stereochemical Quality of Models

In addition to the global accuracy of protein models, we also assess their local stereochemical quality as reported by PROCHECK [60]. Table 2 shows that the stereochemical quality of *e*Thread models, particularly those constructed by *e*Thread/Modeller, is quite high and very close to crystal structures. For example, only 5% less residues are assigned to the most favored regions on the Ramachandran map for the top-ranked models. Top-ranked *e*Thread/TASSER-Lite models are ∼15% worse than these built by *e*Thread/Modeller, suggesting that the former may require more rigorous local structure refinement. Furthermore, in both cases, the top-ranked models typically have higher stereochemical quality than those at lower ranks. Finally, both procedures, *e*Thread/Modeller and *e*Thread/TASSER-Lite, systematically produce models whose quality is notably higher than that obtained by a single-template approach, PSI-BLAST/Nest as well as the standard version of TASSER-Lite, see Table 2.

**Table 2:** Stereochemical quality of protein models.

Region^a^	Crystal	PSI-BLAST/Nest	TASSER-Lite	*e*Thread/Modeller		*e*Thread/TASSER-Lite	
				*Rank 1*	*Rank 2–10*	*Rank 1*	*Rank 2–10*
core	88.2% ±7.4	64.6% ±17.6	63.9% ±9.3	83.4% ±6.7	73.3% ±9.9	68.8% ±9.7	66.6% ±9.6
allow	10.8% ±6.2	20.9% ±8.2	22.2% ±5.7	11.5% ±4.4	17.1% ±5.6	20.2% ±6.3	21.1% ±6.3
gener	0.7% ±1.4	9.7% ±6.7	6.7% ±2.8	2.8% ±1.9	5.3% ±3.0	5.4% ±2.6	5.9% ±2.7
disall	0.3% ±0.7	4.8% ±3.9	7.2% ±2.9	2.3% ±1.6	4.3% ±2.6	5.7% ±2.5	6.3% ±2.6

Models constructed by *e*Thread/Modeller and *e*Thread/TASSER-Lite are compared to crystal structures as well as models built by a simple single-template approach, PSI-BLAST/Nest and a standard version of TASSER-Lite. The quality is assessed by the percentage of residues assigned to different regions of the Ramachandran map by PROCHECK.

a According to PROCHECK classification: core – most favored regions, allow – additional allowed regions, gener – generously allowed regions, disall – disallowed regions.

### Model Quality Assessment

**Figure 7:**
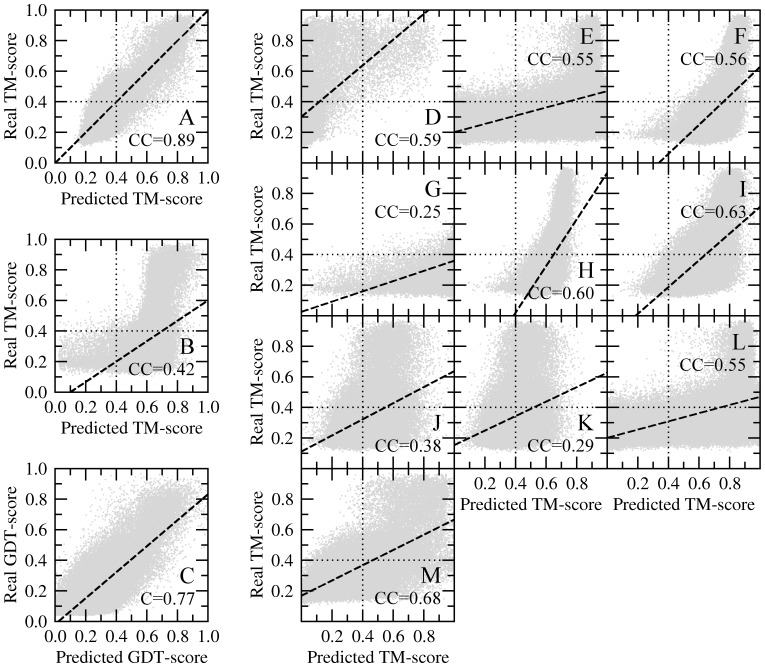
Quality assessment by *e*Rank/Modeller. Three plots on the left show the correlation between the real TM-score of models built by *e*Thread/Modeller and the TM-score estimated by (**A**) *e*Rank, (**B**) SELECTpro and (**C**) APOLLO. For APOLLO, GDT-score is used instead of TM-score. Individual scoring components of *e*Rank are shown on the right: (**D**) *Fraction*, (**E**) *eThread*, (**F**) *DOPE*, (**G**) *GA341*, (**H**) *dFire*, (**I**) *PSIPRED*, (**J**) *SecStr*, (**K**) *Burial*, (**L**) *Alignment*, and (**M**) *Coverage*. In **A**, **B** and **D–M**, dotted lines delineate the TM-score statistical significance threshold.

**Figure 8:**
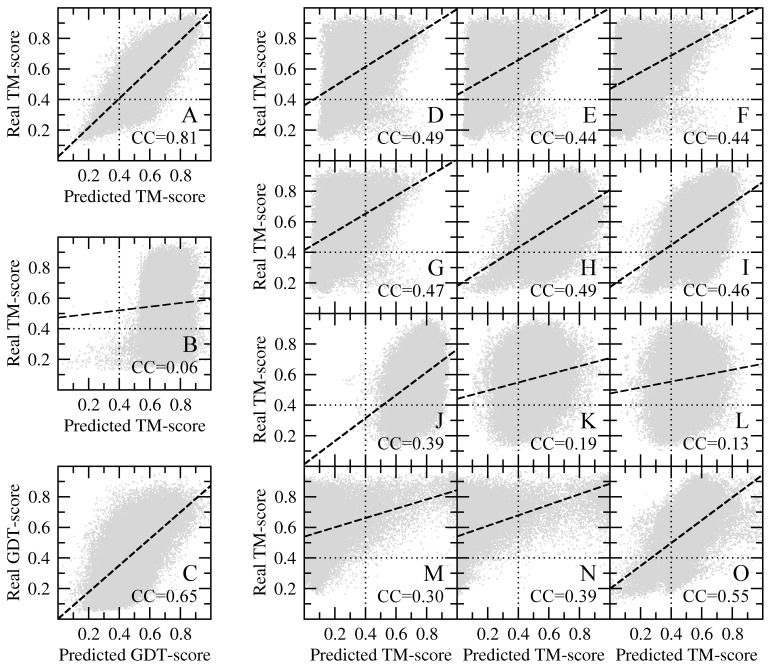
Quality assessment by *e*Rank/TASSER-Lite. Three plots on the left show the correlation between the real TM-score of models built by *e*Thread/TASSER-Lite and the TM-score is estimated by (**A**) *e*Rank, (**B**) SELECTpro and (**C**) APOLLO. For APOLLO, GDT-score is used instead of TM-score. Individual scoring components of *e*Rank are shown on the right: (**D**) *TM-score*, (**E**) *eThread*, (**F**) *Alignment*, (**G**) *Coverage*, (**H**) *DOPE*, (**I**) *dFire*, (**J**) *PSIPRED*, (**K**) *SecStr*, (**L**) *Burial*, (**M**) *TASSER-Lite^fract^*, (**N**) *TASSER-Lite^dens^*, and (**O**) *TASSER-Lite^ene^*. In **A**, **B** and **D–O**, dotted lines delineate the TM-score statistical significance threshold.

A modern structure modeling protocol also requires a reliable system to estimate the modeling confidence, which is often called quality assessment. *e*Rank uses machine learning models appropriate for regression problems to provide this functionality. For a given model, the confidence corresponds to the estimated TM-score to native. Figure 7 shows correlation plots for the top five models constructed by *e*Thread/Modeller. The Pearson correlation coefficient (CC) is used to measure the strength of a linear dependence between the predicted and real TM-score values. CC of 0.89 produced by *e*Rank/Modeller (Figure 7A) is much higher than the individual scoring functions, e.g. *Coverage* (0.68), *PSIPRED* (0.63), *dFire* (0.60) or *DOPE* (0.56). *e*Rank/TASSER-Lite also provides very reliable confidence estimates with a CC of 0.81 (Figure 8), despite the higher density of good models with a TM-score of ≥0.4. Here, the most accurate individual scoring functions, *TASSER-Lite^ene^*, *TM-score* and *DOPE* are notably less accurate with the CC of 0.55, 0.49 and 0.49, respectively.

In both cases, the CC between predicted and real TM-score values for *e*Rank is significantly higher than that obtained by an alternate model quality assessment method, SELECTpro [53], which produces the CC of 0.42 (Figure 7B) and 0.06 (Figure 8B) for *e*Thread/Modeller and *e*Thread/TASSER-Lite models, respectively. A common feature of structure-based methods, such as SELECTpro, DOPE or GA341 is that these algorithms typically recognize good models, but also assign high scores to non-native conformations, which are of acceptable stereochemical quality, e.g. Figure7B, 7E and 7F. In addition to SELECTpro, we also compare *e*Rank to APOLLO [54] using a single-structure mode. Here, we switch to GDT-score, which is the default scoring function used by this algorithm. We note that the real GDT-scores calculated for *e*Thread/Modeller and *e*Thread/TASSER-Lite models correlate very well with the corresponding TM-scores (CC of 0.93 and 0.90, respectively). APOLLO builds more accurate estimates of the global structure quality than SELECTpro and all individual scoring functions. For models constructed by *e*Thread/Modeller and *e*Thread/TASSER-Lite, the CC is 0.77 and 0.65, respectively (see Figures 7C and 8C). Nonetheless, *e*Rank still gives 12–16% higher correlation than APOLLO; thus the scoring function implemented in *e*Rank clearly provides a robust system for the a priori estimate of model divergence from the native conformation.

### eThread Webserver and Datasets

*e*Thread webserver, datasets and modeling results are available for non-commercial users under the terms of GNU General Public License at http://www.brylinski.org/ethread. The webserver allows users to submit amino acid sequences 50–600 residues in length, select the modeling protocol (either *e*Thread/Modeller or *e*Thread/TASSER-Lite) and download the results as well as visualize them directly on the website using OpenAstexViewer [61]. The webserver was designed to be user-friendly and accessible using a Java-enabled web browser and any operating system.

## Discussion

Template-based modeling is currently the most accurate, and consequently the most commonly used, approach for protein structure prediction. The best methods in this area frequently employ meta-threading to identify template structures in available databases, such as PDB [14] and to construct target-to-template alignments. A popular technique used in the development of meta-threading approaches is a gateway approach, which queries several publicly available servers, collects the results and generates consensus predictions [62], [63], [64]. For example, a neural-network approach that combines predictions from six webservers was demonstrated to increase the accuracy of fold recognition by 8–10% [21]. Nevertheless, it was pointed out that the gateway approach may result in unexpected delays and possibly inconsistent results as a consequence of shutting down remote machines, frequent updates and modifications of algorithms used remotely [12]. Thus, a meta-approach set up and maintained in a local environment appears as the most steady, robust and desirable solution.

In this work, we describe the development, optimization and large-scale benchmarking of *e*Thread, a machine learning-based method, which integrates ten state-of-the-art threading/fold recognition algorithms in a local environment to carry out fully automated template-based protein structure modeling. Excluding closely related templates from the modeling process, we evaluate the performance of *e*Thread in template identification, the construction of threading alignments and inter-residue contact prediction. We demonstrate that *e*Thread generates high-quality structural data that can be effectively used to build reliable protein models using available structure assembly algorithms. *e*Thread extensively uses various machine learning techniques to make highly accurate predictions. It has been demonstrated that statistical machine learning effectively utilizes a set of features extracted using general-purpose alignment tools for template ranking; here, sequence profile-profile and profile-structural-profile scores are the most informative [65], [66]. A model based on Support Vector Machines was also successfully applied to estimate the significance of individual target-to-template alignments with a notable improvement over more standard measures such as Z-score or E-value [67]. In our benchmarks, *e*Thread detects significantly more templates than any single-threading algorithm while maintaining a low false-positive rate.

Next to template identification, the construction of corresponding target-to-template alignments is critical to produce high-quality protein models. Alignments generated by different methods can confidently cover different regions of a target sequence, thus the consensus alignment may result in a significant global improvement. Better-aligned parts recognized in a set of alignments generated by different methods can be combined into a unique solution, which is typically more accurate than any of the individual alignments [68], [69]. Here, we developed a machine learning variant of this approach, which applies a Bayesian Classifier to meta-threading alignments to construct a probability-based scoring matrix, which is subsequently used in a traditional Needleman-Wunsch DP. The accuracy of this algorithm is comparable to the best individual alignment method for easy targets, but outperforms other methods in more difficult cases.

Furthermore, we developed *e*Contact, a new machine learning-based method for inter-residue contact prediction that takes advantage of accurately identified templates, good quality target-to-template alignments and a knowledge-based statistical pair potential to recover native contacts at a very low false positive rate. In contact prediction, applying non-linear models, such as support vector machines, frequently outperforms many of the simple majority voting methods [52], [70]. In addition to protein structure modeling, the predicted inter-residue contacts are also useful for the estimation of protein folding rates [71].

To assembly three-dimensional models of the target proteins using *e*Thread templates and alignments, we tested two popular structure modeling algorithms: Modeller [22] and TASSER-Lite [23]. Both programs perform comparably well and generate models, which are correct at the fold level, for >80% of the targets. However, significantly less of the constructed models (40–50%) are of a very high quality, which would be considered accurate at the family level [72]. Here, the upper bound estimated using structure alignments is ∼75%, which suggest that further advances in threading methodologies could bring about 25% improvement in low-homology template-based modeling. Generating near-experimental quality structural models using “twilight zone” templates [73] would therefore require different modeling techniques, such as all-atom refinement [74], [75], [76]. A gray-bricked area in Figure 6 points up a substantial room for potential improvement using structure refinement, which increases with the requirement of protein models to be closer to experimental structures. In this study, we employ a very simple procedure for all-atom refinement using molecular mechanics, which mostly optimizes side chain geometries and removes atom clashes. Using more advanced refinement could yield additional improvement in model quality, particularly in the high TM-score regime.

Many state-of-the-art protein structure prediction algorithms often generate a set of possible models for a given target. This is particularly common in low-homology multiple-template modeling. Thus, there is a need to select the most native-like conformation from a pool of constructed models. To address this issue, we developed *e*Rank, which employs support vector machines for ranking problems to provide a very robust approach to model ranking. In addition, *e*Rank also produces reliable confidence estimates, which correlate well with the actual model quality. This is particularly important for the use of modeled structures in structure-based function annotation. For example, in ligand and macromolecular docking, the selection of modeling protocol strongly depends on the quality of the target protein structures. While all-atom docking is applicable to high-quality receptor structures [77], [78], using low-to-moderate quality protein models often requires different algorithms, such as low-resolution modeling [79], [80], to provide confident annotations.

### Conclusions

We present a suite of programs: *e*Thread, *e*Contact and *e*Rank, which build on meta-threading and conduct fully automated template-based protein structure modeling. This meta-approach extensively uses machine learning techniques to generate good quality protein models even in the presence of only distantly homologous template structures and offers a reliable system for confidence estimates. Comparative benchmarks show that it outperforms other methods for inter-residue contact prediction, template-based structure modeling as well as model selection and quality assessment. *e*Thread is freely available to the academic community through a user-friendly webserver at http://www.brylinski.org/ethread.

## Supporting Information

Text S1 **Calculation of the burial score and secondary structure preferences for model ranking and confidence estimates.**
(PDF)
