# Supplementary material for: eThread: A Highly Optimized Machine Learning-Based Approach to Meta-Threading and the Modeling of Protein Tertiary Structures
Source: PLoS One. 2012 Nov 21;7(11):e50200. doi: 10.1371/journal.pone.0050200 (PMC3503980; doi:10.1371/journal.pone.0050200)
Supplement: Text S1 — Calculation of the burial score and secondary structure preferences for model ranking and confidence estimates. (PDF) [file pone.0050200.s001.pdf]

***e*Thread: A highly optimized machine learning-based approach to meta-threading and the modeling of protein tertiary structures**

Supporting Information

Burial score considers a 3-state residue classification: *S* (surface), *M* (middle layer) and *I* (interior). A protein structure is placed in an ellipsoid, whose size is calculated from mass-weighted principal axes [1,2]. Each residue is assigned to a particular class based on the distances between its Cα atom, the ellipsoid center and the ellipsoid surface. If the distance to the surface is twice as long as to the center, a residues is considered buried (*I*), if the distance is twice as short it is considered surface (*S*). Remaining residues are assigned a mid-layer class *M*. First, we calculated the composition of the amino acid of type *A* within a state *B* in the non-redundant CATH database [3] as follows:

$$C_{A,B}^{bur} = N_{A,B}^{bur} \Big/ \sum_{i=1}^{20} N_{i,B}^{bur} \quad \text{Eq. 1}$$

where $N_{i,B}^{bur}$ is the number of amino acids of type *i* within the state *B*.

The normalized burial composition for a given amino acid *A* is defined as its composition in the particular state *B* (calculated from the structure) divided by its frequency of occurrence, f_A, in the dataset:

$$S_{A,B}^{bur} = \frac{C_{A,B}^{bur}}{f_A} \quad \text{Eq. 2}$$

For a given protein of length *n*, the total burial score, *Burial*, is calculated as the burial score averaged over all residues:

$$Burial = \frac{1}{n}\sum_{i=1}^{n} S_{i,B}^{bur} \qquad \text{Eq. 3}$$

Secondary structure score is calculated in a similar fashion using a 7-state classification by STRIDE [4]: H – α-helix, G – 3-10 helix, I – π-helix, E – extended conformation, B – isolated bridge, T – turn and C – coil. Similarly to the burial score, we calculated the composition of the amino acid of type A within a secondary structure D in the non-redundant CATH database, $C_{A,D}^{\sec}$. The total secondary structure score, *SecStr*, is calculated as the normalized secondary structure composition, $S_{A,D}^{\sec}$, averaged over all residues:

$$SecStr = \frac{1}{n}\sum_{i=1}^{n} S_{i,D}^{\sec} \qquad \text{Eq. 4}$$

References

1. Browner MF, Fauman EB, Fletterick RJ (1992) Tracking conformational states in allosteric transitions of phosphorylase. Biochemistry 31: 11297-11304.
2. Brylinski M, Skolnick J (2008) What is the relationship between the global structures of apo and holo proteins? Proteins 70: 363-377.
3. Orengo CA, Michie AD, Jones S, Jones DT, Swindells MB, et al. (1997) CATH--a hierarchic classification of protein domain structures. Structure 5: 1093-1108.
4. Frishman D, Argos P (1995) Knowledge-based protein secondary structure assignment. Proteins 23: 566-579.
